# Salidroside Suppresses HUVECs Cell Injury Induced by Oxidative Stress through Activating the Nrf2 Signaling Pathway

**DOI:** 10.3390/molecules21081033

**Published:** 2016-08-09

**Authors:** Yao Zhu, Ya-Jie Zhang, Wei-Wei Liu, Ai-Wu Shi, Ning Gu

**Affiliations:** 1 First College of Clinical Medicine, Nanjing University of Traditional Chinese Medicine, Nanjing 210023, China; zy_njtcm@126.com (Y.Z.); cheeseweiweiliu@126.com (W.-W.L.); 2 Nanjing Hospital of Traditional Chinese Medicine, Nanjing 210001, China; zhangyajie_jack@126.com; 3 Nanjing Maternity and Child Health Care Hospital, Nanjing 210004, China

**Keywords:** salidroside, oxidative stress, nuclear factor E2-related factor 2, heme oxygenase-1, NAD(P)H dehydrogenase (quinone1), human umbilical vein endothelial cells

## Abstract

Oxidative stress plays an important role in the pathogenesis of cardiovascular diseases. Salidroside (SAL), one of the main effective constituents of *Rhodiola rosea*, has been reported to suppress oxidative stress-induced cardiomyocyte injury and necrosis by promoting transcription of nuclear factor E2-related factor 2 (Nrf2)-regulated genes such as heme oxygenase-1 (HO-1) and NAD(P)H dehydrogenase (quinone1) (NQO1). However, it has not been indicated whether SAL might ameliorate endothelial injury induced by oxidative stress. Here, our study demonstrated that SAL might suppress HUVEC cell injury induced by oxidative stress through activating the Nrf2 signaling pathway. The results of our study indicated that SAL decreased the levels of intercellular reactive oxygen species (ROS) and malondialdehyde (MDA), and improved the activities of superoxide dismutase (SOD) and catalase (CAT), resulting in protective effects against oxidative stress-induced cell damage in HUVECs. It suppressed oxidative stress damage by inducing Nrf2 nuclear translocation and activating the expression of Nrf2-regulated antioxidant enzyme genes such as HO-1 and NQO1 in HUVECs. Knockdown of Nrf2 with siRNA abolished the cytoprotective effects against oxidative stress, decreased the expression of Nrf2, HO-1, and NQO1, and inhibited the nucleus translocation of Nrf2 in HUVECs. This study is the first to demonstrate that SAL suppresses HUVECs cell injury induced by oxidative stress through activating the Nrf2 signaling pathway.

## 1. Introduction

Oxidative stress is defined as an excessive production of reactive oxygenated species that cannot be counteracted by the action of antioxidants, but also as a perturbation of cell redox balance [1,2]. Excessive productions of reactive oxygen species (ROS), such as O_2_^−^ (superoxide radical), OH (hydroxyl radical) and H_2_O_2_ (hydrogen peroxide), can cause base damage, strand breaks in DNA, as well as disruptions in normal mechanisms of cellular signaling [3,4].

Endothelial dysfunction plays a key role in cardiovascular diseases. The imbalance between the production of ROS and their effective removal by non-enzymatic and enzymatic antioxidant systems could induce endothelial dysfunction through inducing injury, apoptosis and necrosis of endothelial cells, promoting accumulation of macrophage-derived cytokines, such as tumor necrosis factor (TNF), and increasing the permeability of endothelium [5,6,7]. Clinical studies have reported that oxidative stress may contribute not only to endothelial dysfunction, but also to coronary artery disease activity, increasing the risk of cardiovascular events in patients with coronary artery disease [8]. Thus the development of an effective antioxidant strategy to reduce oxidative stress in the progress of endothelial dysfunction may represent fruitful avenues for biological investigation and for the identification of new therapeutic targets.

Nuclear factor-erythroid 2-related factor 2 (Nrf2) is a basic leucine zipper (bZIP) protein that regulates the expression of antioxidant proteins that protect against environmental oxidative stress [9,10]. Normally Nrf2 is degraded in the cytoplasm by Kelch like-ECH-associated protein 1 (Keap1)-Cullin3 ubiquitination system. Oxidative stress or electrophilic stress disrupts the Keap1-Cullin3 system, and allows Nrf2 to translocate into the nucleus [11,12]. In the nucleus, Nrf2 binds to the antioxidant response element (ARE), resulting in the transcription of antioxidative genes such as heme oxygenase-1 (HO-1) [13], NAD(P)H quinone oxidoreductase 1 (NQO1) [14], glutathione S-transferase (GST) [15], UDP-glucuronosyltransferases (UDPGTs) [16], aflatoxin aldehyde reductase (AAR) [17], superoxide dismutase (SOD) [18], catalase (CAT) [19], gamma-glutamine cysteine synthase (g-GCS) [15], glutathione reductase (GR) [20], and thioredoxin reductase (TR) [21].

**Figure 1:**
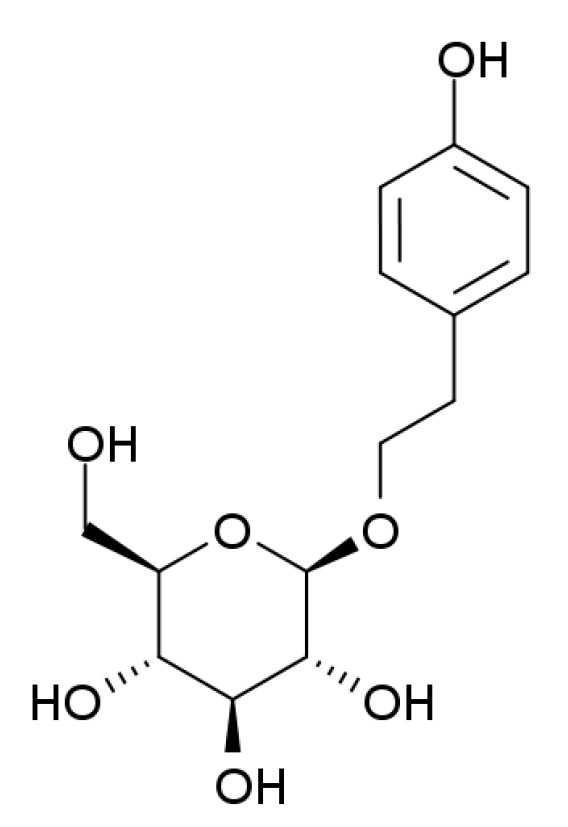
Chemical structure of salidroside (SAL), CAS number: 10338-51-9.

*Rhodiola rosea* has long been used in Chinese medicine in clinical practice for the treatment of various diseases because of its antioxidant properties. Salidroside (SAL, Figure 1), one of the main effective constituents of *Rhodiola rosea*, possesses a wide range of pharmacological activities such as antioxidant, anti-inflammatory, anticancer, cardioprotective, and neuroprotective effects [22,23,24,25,26,27]. Various studies have demonstrated that SAL exerts a protective effect against cellular injury and apoptosis by altering signal transduction in cells. For example, it was reported that SAL could alleviate ethanol-induced acute gastric ulcer, H_2_O_2_-induced gastric epithelial cell damage, enhance antioxidant activity, and inhibit the overproduction of pro-inflammatory cytokines through the mitogen-activated protein kinase (MAPK)/nuclear factor kappa B (NF-κB) pathway [28]. A previous study indicated that salidroside might be a functional chemopreventative agent that ameliorates cadmium-induced cytotoxicity in hepatocytes via gap junction intercellular communication (GJIC) and MAPK pathways [29]. Furthermore, it was demonstrated that salidroside exerts angiogenic and cytoprotective effects on human bone marrow-derived endothelial progenitor cells via Akt/ mammalian target of rapamycin (mTOR)/p70S6K and MAPK signalling pathways [30].

In this study, we investigated the ability of SAL to prevent cell injury of human umbilical vein endothelial cells (HUVECs) induced by oxidative stress. Moreover, the intracellular ROS, malondialdehyde (MDA), SOD and CAT were evaluated to identify SAL protective function and the expression of Nrf2, HO-1, NQO1 and nuclear translocation of Nrf2 were evaluated to clarify the molecular mechanisms responsible for its preventive effects against oxidative stress.

## 2. Results

### 2.1. SAL Prevents H_2_O_2_-Induced Cell Injury

**Figure 2:**
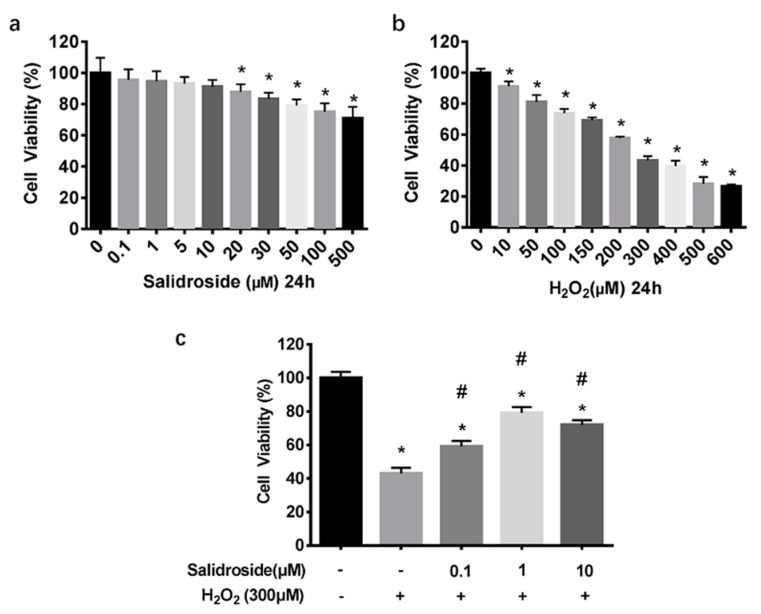
SAL prevents H_2_O_2_-induced cell injury. Cell viability was determined by MTT assay, and the viability of untreated HUVECs was taken as 100%. (**a**) HUVECs were incubated with SAL (0.1–500 μM) for 24 h. (**b**) HUVECs were treated with various concentrations of H_2_O_2_ (0–600 μM) for 24 h. (**c**) HUVECs were treated with SAL (0.1, 1, 10 μM) for 24 h before being treated with H_2_O_2_ (300 μM) for another 24 h. Data are presented as mean ± SD values of three independent experiments. * *p* < 0.05 vs. control. ^#^ *p* < 0.05 vs. H_2_O_2_-treated HUVECs.

To determine the cytotoxic potential of SAL, its effects on cell viability of HUVECs was evaluated. Incubation with 0.1–10 μM SAL for 24 h didn’t affect cell viability, whereas higher concentrations (20–500 μM) reduced viability significantly (Figure 2a). Therefore, SAL concentrations of 0.1, 1, and 10 μM were selected for subsequent experiments. MTT assays also showed that incubation with 300 μM H_2_O_2_ for 24 h could reduce viability of HUVECs to 43.40% ± 2.41% (Figure 2b). Next, we examined the protective effects of SAL against H_2_O_2_-induced cell damage. HUVECs were pretreated with SAL (0.1, 1, and 10 μM) for 24 h and then exposed to H_2_O_2_ (300 μM) for another 24 h. Results demonstrated that incubating HUVECs with SAL (0.1, 1, and 10 μM) for 24 h antagonized the effects of 300 μM H_2_O_2_ on cell viability, and pretreatment with 1 μM SAL increased cell viability to 79.07% ± 3.13%, which was better than the others (Figure 2c).

### 2.2. SAL Inhibits H_2_O_2_-Induced Oxidative Stress in HUVECs

**Figure 3:**
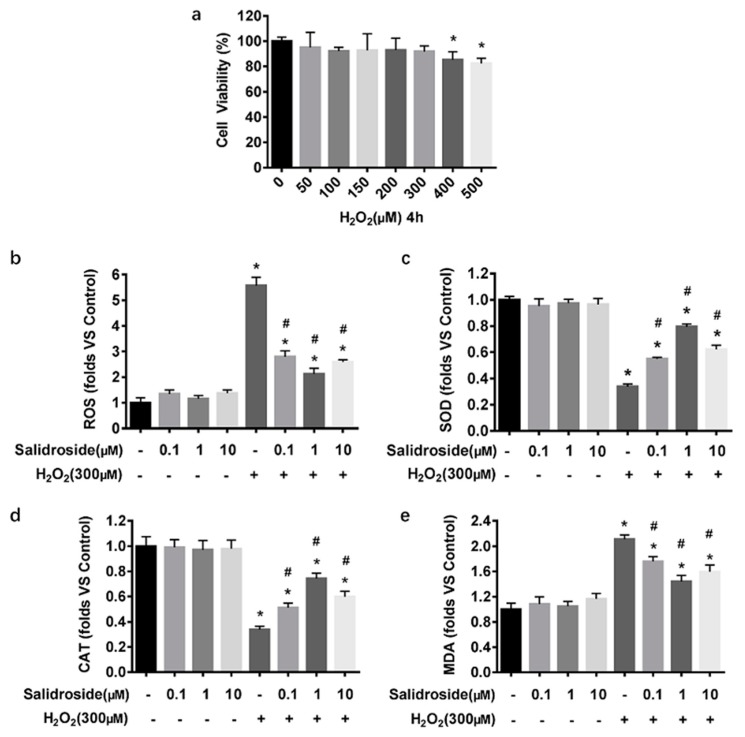
SAL reduces H_2_O_2_-induced ROS production and MDA formation and increases the activities of antioxidant enzymes in HUVECs. (**a**) HUVECs were treated with various concentrations of H_2_O_2_ (0–500 μM) for 4 h, and cell viability was determined by MTT assay. (**b**–**e**) HUVECs were incubated without any intervention (control), SAL (0.1, 1, 10 μM) for 24 h, or 300 μM H_2_O_2_ for 4 h, or 0.1, 1, 10 μM SAL for 24 h followed by 300 μM H_2_O_2_ for an additional 4 h. (**b**) Intercellular ROS levels: Intercellular ROS levels were determined by DCFH-DA fluorescence. After various treatment, the cells were stained with 10 μM DCFH-DA in serum free medium for 30 min in the dark. Fluorescence intensity was recorded using a Synergy^TM^ 4 Multi-Mode Microplate Reader at an excitation wavelength of 488 nm and an emission wavelength of 525 nm. (**c**–**e**) SOD, CAT activities, and MDA content: SOD, CAT activities, and MDA content were measured using respective assay kits. Data are presented as mean ± SD values of three independent experiments. * *p* < 0.05 vs. control. ^#^ *p* < 0.05 vs. H_2_O_2_-treated HUVECs.

MTT assays showed that incubation with 50–300 μM H_2_O_2_ for 4 h didn’t affect the viability of HUVECs, whereas higher concentrations (400, 500 μM) reduced viability of these cells significantly (Figure 3a). Therefore, oxidative stress was induced by addition of H_2_O_2_ (300 μM) to the culture medium for 4 h. In order to evaluate whether SAL protected HUVECs from oxidative stress induced by H_2_O_2_, the effects of pre-treatment with SAL on the levels of intracellular SOD, CAT, MDA, and ROS were determined. We measured ROS and MDA as indicators of cellular oxidative state, and SOD and CAT as indicators of enzymatic antioxidant state. Results showed that 300 μM H_2_O_2_ significantly increased cellular ROS and MDA levels and decreased cellular SOD and CAT levels (all *p* < 0.05, Figure 3b–e). At 0.1, 1, and 10 μM, SAL alone didn’t affect levels of ROS, MDA, SOD, and CAT in the HUVECs significantly, but abolished the H_2_O_2_-induced ROS and MDA production increasing, SOD and CAT levels decreasing significantly (all *p* < 0.05, Figure 3b–e). Furthermore, the effects of pretreatment with 1 μM SAL on inhibiting H_2_O_2_-induced oxidative stress in HUVECs were better than the others.

### 2.3. SAL Stimulated Nrf2, HO-1, and NQO1 Expression in H_2_O_2_-Treated HUVECs

**Figure 4:**
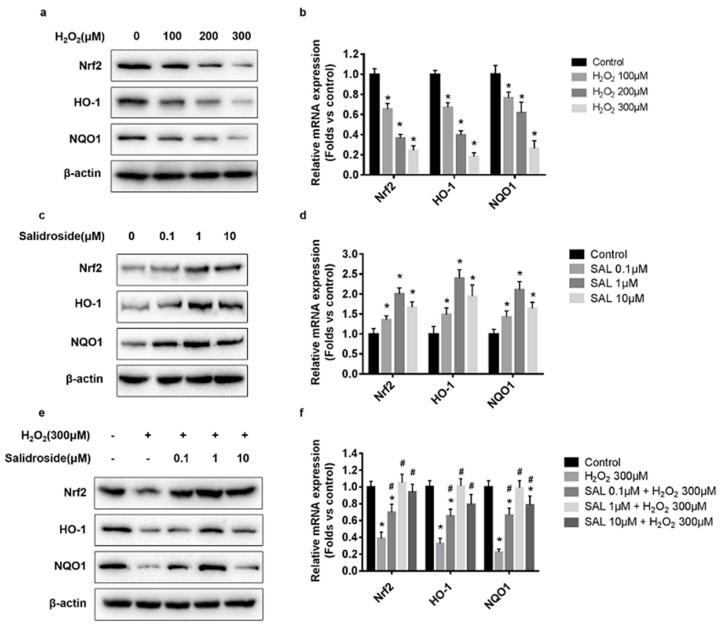
Effects of SAL and H_2_O_2_ on protein and mRNA expression levels of Nrf2, HO-1, and NQO1 in HUVECs. (**a**,**b**) Expression of Nrf2, HO-1, and NQO1 in H_2_O_2_-treated HUVECs. HUVECs were incubated with H_2_O_2_ (100–300 μM) for 4 h, relative protein levels were measured using Western blotting, and relative mRNA levels were determined using RT-PCR. (**c**,**d**) Expression of Nrf2, HO-1, and NQO1 in SAL-treated HUVECs. HUVECs were incubated with SAL (0.1, 1, 10 μM) for 24 h, and total cell lysates were subjected to Western blotting and RT-PCR. (**e**,**f**) Effects of SAL on Nrf2, HO-1, and NQO1 expression in H_2_O_2_-treated HUVECs. HUVECs were incubated with SAL (0.1, 1, 10 μM) for 24 h, followed by 300 μM H_2_O_2_ for an additional 4 h, relative protein levels were measured using Western blotting, and relative mRNA levels were determined using RT-PCR. The results of RT-PCR were normalized to β-actin, and expressed as fold change to control. The values represent the means ± SD of triplicate experiments. * *p* < 0.05 vs. control. ^#^ *p* < 0.05 vs. H_2_O_2_-treated HUVECs.

To determine the effects of H_2_O_2_ on the expression of Nrf2, HO-1, and NQO1, HUVECs were incubated with H_2_O_2_ (100–300 μM) for 4 h, and relative mRNA and protein levels of Nrf2, HO-1, and NQO1 were measured by RT-PCR and western blotting. The western blotting results showed that exposure of HUVECs to 100–300 μM H_2_O_2_ for 4 h significantly decreased Nrf2, HO-1, and NQO1 protein expression in a dose-dependent manner (Figure 4a). Similarly, results of RT-PCR showed that incubation with 100–300 μM H_2_O_2_ for 4 h dose-dependently decreased the mRNA levels of Nrf2, HO-1, and NQO1 (Figure 4b).

Next, we tested the effects of SAL on the expression of Nrf2, HO-1, and NQO1. HUVECs were incubated with SAL (0.1–10 μM) for 24 h, and relative protein and mRNA levels of Nrf2, HO-1, and NQO1 were measured using western blotting and RT-PCR. Evaluation of Nrf2, HO-1, and NQO1 by western blotting showed that exposure of HUVECs to 0.1–10 μM SAL strongly induced Nrf2, HO-1, and NQO1 protein expression (Figure 4c). Similarly, RT-PCR results shown in Figure 4d demonstrate that pretreatment with SAL at 0.1–10 μM for 24 h significantly increased the mRNA levels of Nrf2, HO-1, and NQO1. Moreover, incubation with 1 μM SAL induced the expression of Nrf2, HO-1, and NQO1, more strongly than the others.

Finally, to verify whether SAL could rescue the decrease of Nrf2, HO-1, and NQO1 expression under 300 μM H_2_O_2_ treatment, HUVECs were incubated with SAL (0.1, 1, 10 μM) for 24 h, followed by 300 μM H_2_O_2_ for an additional 4 h, and relative protein and mRNA levels of Nrf2, HO-1, and NQO1 were measured using western blotting and RT-PCR. Western blotting and RT-PCR results showed that pretreatment with 0.1–10 μM SAL resulted in a dramatic increase in both protein and mRNA expression of Nrf2, HO-1, and NQO1 (Figure 4e,f). Moreover, incubation with 1 μM SAL stimulated Nrf2, HO-1, and NQO1 expression in H_2_O_2_-treated HUVECs more strongly than others.

### 2.4. SAL Induced Nucleus Accumulation of Nrf2 in HUVECs

Normally, Nrf2 is kept in the cytoplasm by Keap1. Once stimulated, Nrf2 translocates into the nucleus, binds to the promoter regions of ARE, and activates its target genes, such as HO-1 and NQO1 [11,12,13]. Therefore, to investigate whether pretreatment with SAL induces Nrf2 nucleus translocation in HUVECs, we examined the protein expression and subcellular location of Nrf2 in HUVECs.

**Figure 5:**
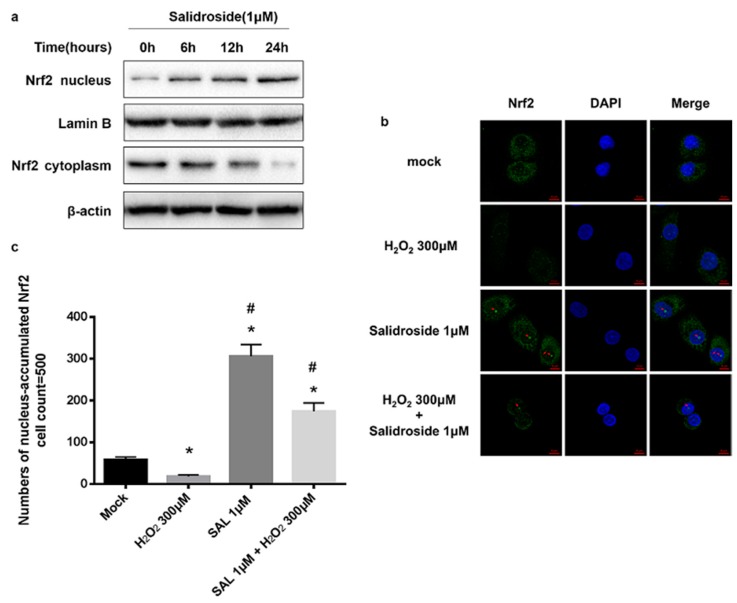
Effects of SAL on Nrf2 nucleus accumulation. (**a**) Time effects of SAL on Nrf2 nuclear translocation. HUVECs were incubated with 1 μM SAL for 6, 12, 24 h, and nuclear and cytosolic fractions were isolated according the manufacturer’s instruction, as described in the Experimental Section. Protein levels of Nrf2 in nucleus and in cytoplasm were measured by western blotting. (**b**) Immunofluorescence staining of Nrf2. HUVECs were incubated with 1 μM SAL for 24 h, or 300 μM H_2_O_2_ for 4 h, or 1 μM SAL for 24 h and 300 μM H_2_O_2_ for a further 4 h. Nrf2 was stained green with Alexa Fluor® 488. The nucleus was stained with DAPI, and the merge represents the combined image of Nrf2 fluorescence and nuclear staining. The same results were obtained in three independent experiments. (**c**) Pooled data of nucleus-accumulated Nrf2. For each group, we counted the numbers of nucleus-accumulated Nrf2 in different visual fields for 500 cells. Data was presented as mean ± SD, *n* = 500, * *p* < 0.05 vs. control. ^#^ *p* < 0.05 vs. H_2_O_2_-treated HUVECs.

HUVECs were incubated with 1 μM SAL for 6, 12, 24 h, and nuclear and cytosolic fractions were separated according manufacturer’s instruction, as described in the Experimental Section. Protein levels of Nrf2 in nucleus and in cytoplasm were measured by western blotting. As shown in Figure 5a, western blot analysis of the nuclear fraction of SAL-treated HUVECs demonstrated a significant increase in Nrf2 protein levels in a time-dependent manner (Figure 5a).

HUVECs were incubated with 1 μM SAL for 24 h, or 300 μM H_2_O_2_ for 4 h, or 1 μM SAL for 24 h and 300 μM H_2_O_2_ for a further 4 h, and then the immunofluorescence staining of Nrf2 in HUVECs were performed. Results of immunofluorescence staining showed incubation with H_2_O_2_ inhibited Nrf2 from accumulating in nucleus, whereas incubation with 1 μM SAL induced nucleus accumulation of Nrf2, which could rescue Nrf2 suppression induced by H_2_O_2_ in HUVECs (Figure 5b,c).

### 2.5. Knockdown of Nrf2 with siRNA Decreased the Expression of Nrf2, HO-1, and NQO1, and Inhibited the Nucleus Translocation of Nrf2 in HUVECs

**Figure 6:**
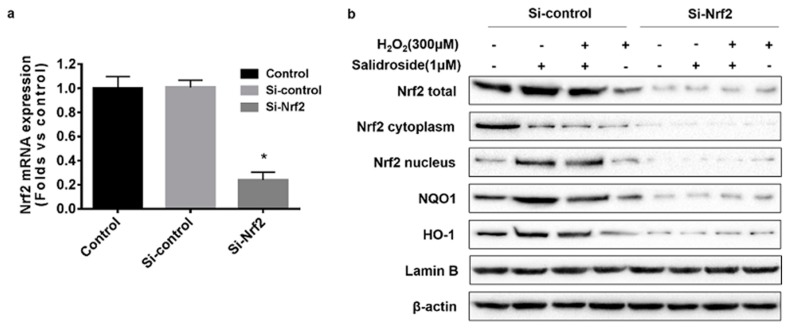
Effects of SAL on the expression of HO-1, and NQO1 in Nrf2 knockdown HUVECs. (**a**) HUVECs were transiently transfected with Nrf2 siRNA and scrambled siRNA, and incubated for 48 h. Nrf2 mRNA expression was determined using RT-PCR. The results of RT-PCR were normalized to β-actin, and expressed as fold change to control. The values represent the means ± SD of triplicate experiments. * *p* < 0.05 vs. control. (**b**) HUVECs were treated with Nrf2 siRNA and scrambled siRNA. After 48 h, the cells were incubated with 1 μM SAL for 24 h, or 300 μM H_2_O_2_ for 4 h, or 1 μM SAL for 24 h and 300 μM H_2_O_2_ for a further 4 h, and the protein levels of Nrf2 (total, nuclear, and cytoplasmic), HO-1, and NQO1 were determined using western blotting.

To confirm the role of Nrf2 in SAL-induced HO-1 and NQO1 upregulation, we transfected HUVECs with Nrf2 siRNA and scrambled siRNA, and incubated for 48 h before treatment. As shown in Figure 6a, Nrf2 mRNA expression in HUVECs was downregulated to about 23% of mock and si-control, after incubation with Nrf2 siRNA for 48 h. The western blotting results showed silencing Nrf2 expression significantly suppressed SAL-induced HO-1 and NQO1 up-regulation, suggesting induction of HO-1 and NQO1 by SAL is dependent on activation of Nrf2 (Figure 6b).

### 2.6. SAL-Induced Cytoprotective Effects against Oxidative Stress Induced by H_2_O_2_ in HUVECs Are Dependent on Activation of Nrf2

**Figure 7:**
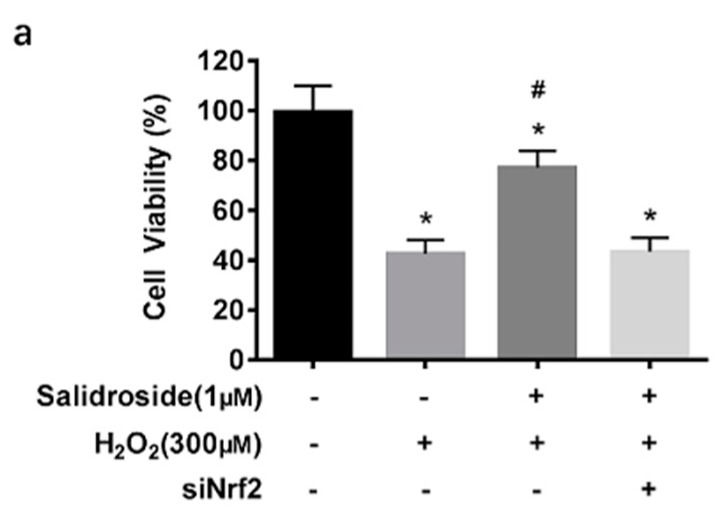

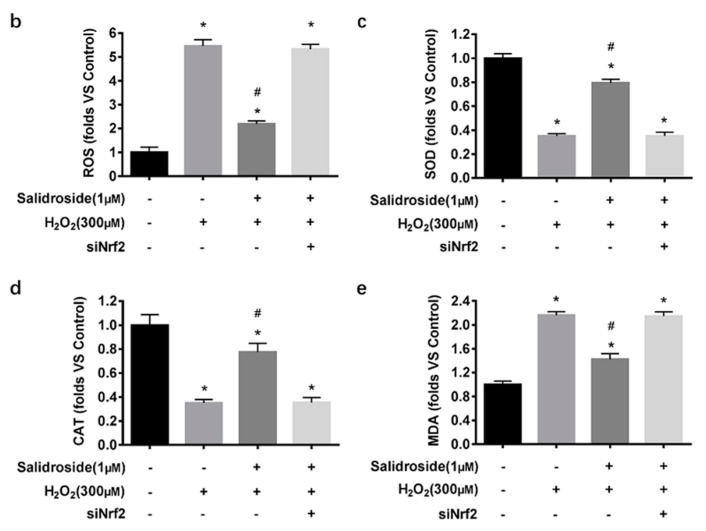
Effects of SAL on H_2_O_2_-induced cytotoxicity and oxidative stress in Nrf2 knockdown HUVECs. (**a**) Cell viability analysis. HUVECs were preincubated with or without Nrf2 siRNA, then treated with 1 μM SAL for 24 h before being treated with 300 μM H_2_O_2_ for another 24 h, or 300 μM H_2_O_2_ for 24 h. Cell viability was determined using MTT assay. (**b**–**e**) HUVECs were preincubated with or without Nrf2 siRNA, then treated with 300 μM H_2_O_2_ for 4 h, or 1 μM SAL for 24 h followed by 300 μM H_2_O_2_ for an additional 4 h. (**b**) Intercellular ROS levels: Intercellular ROS levels were determined by DCF fluorescence. After various treatment, the cells were stained with 10 μM DCFH-DA in serum free medium for 30 min in the dark. Fluorescence intensity was recorded using a Synergy^TM^ 4 Multi-Mode Microplate Reader. (**c**–**e**) SOD, CAT activities, and MDA content: SOD, CAT activities, and MDA content were measured using respective assay kits. Data are presented as mean ± SD values of three independent experiments. * *p* < 0.05 vs. control. ^#^ *p* < 0.05 vs. H2O2-treated HUVECs.

To investigate the role of Nrf2 in SAL-induced cytoprotective effects against oxidative stress induced by H_2_O_2_ in HUVECs, the cells were preincubated with or without Nrf2 siRNA before various treatment, and then cell viability, intercellular ROS levels, SOD, CAT activities, and MDA content were measured. As shown in Figure 7a, silencing Nrf2 expression significantly reduced the cytoprotective effects of SAL. Nrf2 siRNA also eliminated the antioxidant activity (Figure 7b–e).

## 3. Discussion

Excessive production of ROS in mitochondria is an important risk factor of cardiovascular diseases [31,32], and there is growing concern on the antioxidant therapies of scavenging ROS [33]. Endothelial dysfunction is one of the most important risk factors for cardiovascular diseases, and it represents the initial step in the pathogenesis of atherosclerosis. Failure to protect against oxidative stress-induced cellular damage accounts for endothelial dysfunction in the majority of pathophysiological conditions [34]. AREs are specific DNA-promoter sequences that are located at the 5’-terminal ends of the promoter sequences for various phase II detoxification enzymes and antioxidant enzymes. Nrf2 is the most important activator of AREs. Under oxidative stress conditions, Nrf2 dissociates from Keap1, translocates into the nucleus, combines with the Maf protein to form a heterodimer, and recognizes the appropriate ARE sequence. ARE-mediated gene transcription is subsequently activated. This is the Nrf2/Keap1–ARE pathway [9,10,11,12,34]. Various researches indicated that increased nuclear accumulation of Nrf2 and increased transcriptional activities of Nrf2, as well as its downstream genes, effectively protect endothelial cells from cell damage induced by oxidative stress [35,36,37,38].

SAL, as an efficacious antioxidant, has been widely researched. A previous study demonstrated that 200 μM SAL was capable of protecting retinal endothelial cells from apoptosis induced by oxidative stress through increasing the Bcl2/Bax signaling pathway and enzymatic activities of catalase and Mn-SOD [39]. In addition, another research showed that SAL protected HUVECs against H_2_O_2_-induced oxidative injury, and that the potential mechanisms may involve increasing REDD1 expres­sion to prevent the generation of ROS, modulating the expression of HIF-1α and regulating the activation of the PI3K/Akt pathway followed by activating the downstream molecules of mTOR and SP6 to reduce H_2_O_2_-induced apoptosis [40]. Meanwhile, a recent study reported that SAL could protect endothelium against H_2_O_2_-induced cell injury via promoting mitochondrial biogenesis and function, thus preventing the overactivation of oxidative stress-related downstream signaling pathways [41]. Furthermore, it was demonstrated that SAL could suppress oxidative stress-induced pulmonary fibrosis, cardiomyocyte injury and necrosis, and cerebral ischemia/reperfusion injury, by promoting transcription of Nrf2-regulated genes (HO-1 and NQO1), thus decreasing excessive production of ROS and improving mitochondrial function [42,43,44,45,46]. However, it has not been pointed out that whether SAL might ameliorate endothelial injury induced by oxidative stress, through Nrf2 signaling pathway. Therefore, our result demonstrated SAL suppressed HUVECs cell injury induced by oxidative stress through activating the Nrf2 signaling pathway.

H_2_O_2_ is a classical peroxide. H_2_O_2_ modulates endothelial cell function via intricate mechanisms. Ambient production of O_2_^−^, which serves as a progenitor for H_2_O_2_, and subsequently H_2_O_2_ at low levels, maintained by basal activity of endothelial NAD(P)H oxidases, or mitochondrial respiration, were necessary for endothelial cell growth and proliferation [47,48]. However, H_2_O_2_, when produced in large quantities, might modulate different aspects of endothelial function, including endothelial apoptosis, endothelial cytoskeletal reorganization and barrier dysfunction, endothelial inflammatory responses, and endothelium-regulated vascular remodeling. These modulations of endothelial cell function may at least partially underlie H_2_O_2_ contribution to the development of vascular disease [49]. H_2_O_2_ effects on Nrf2 activation seem strongly dependent both on the H_2_O_2_ concentration and incubation time. For example, Covas et al. found that low sustained (12.5 μM, 0–120 min) H_2_O_2_ concentration preferentially triggers de novo Nrf2 synthesis and nuclear translocation, However, higher concentrations (200–400 μM) for a shorter period of time (15 min) or 12.5 μM H_2_O_2_ for a long period of time (240–360 min) decreased Nrf2 expression and nuclear translocation [50]. Fourquet, et al. exposed HeLa cells to 0.2 mM H_2_O_2_ for different time (0–60 min), and the results showed that expression of Nrf2 increased when exposure to 0.2 mM H_2_O_2_ for 0–60 min, and peaked when exposure to 0.2 mM H_2_O_2_ for 10 min, then it decreased in a time-dependent manner. Finally, they concluded that H_2_O_2_, as a short-lived inducer of Nrf2, had a temporary effect on oxidation that caused only moderate Nrf2 stabilization [51]. The same phenomenon was found in another peroxide, PM 2.5. Yang et al. found that low-doses of PM 2.5 (0–100 μg/mL) upregulated Nrf2 and HO-1 expression in HUVECs, whereas high-doses of PM 2.5 (200–400 μg/mL) increased intracellular ROS, decreased cell viability, and the expression of Nrf2 and HO-1 [38]. In our study, we expect make a cell model which H_2_O_2_ suppress the endogenous background oxidative stress pathways, like Nrf2 pathway, aiming to detect whether SAL could rescue these pathways though pre-protection function. So we set a series of H_2_O_2_ concentration and time points to find the appropriate concentration and time which the Nrf2 pathway could be suppressed and the cells were not triggering apoptotic mechanism at the same time (which was shown in Figure 2b and Figure 3a). At last, we found 300 μM, 4 h was the appropriate stimulation to our cell model.

According to our results, we found that incubated with 0–75 μM H_2_O_2_ for 4 h increased the expression of Nrf2, HO-1, and NQO1, and the expression of Nrf2, HO-1, and NQO1 decreased in a time-dependent manner when the concentrations of H_2_O_2_ were more than 100 μM (data not shown here).

The results of our study also found that SAL increased the expression of Nrf2-regulated antioxidant enzyme genes such as HO-1 and NQO1, decreased the levels of intercellular ROS and MDA, and improved the activities of SOD and CAT, resulting in its protective effects against oxidative stress-induced cell damage in HUVECs. SAL also stimulated the expression and nucleus accumulation of Nrf2 in HUVECs. Knockdown of Nrf2 with siRNA abolished the cytoprotective effects against oxidative stress, decreased the expression of Nrf2, HO-1, and NQO1, and inhibited the nucleus translocation of Nrf2 in HUVECs.

In conclusion, this study is the first to demonstrate that SAL suppresses HUVECs cell injury induced by oxidative stress through activating the Nrf2 signaling pathway. SAL/Nrf2-ARE signaling pathway might be a new idea for the prevention or treatment of endothelial dysfunction induced by oxidative stress, but further animal experiments and clinical trials are necessary to confirm the therapeutic effects of SAL in vivo.

## 4. Experimental Section

### 4.1. Reagents

SAL (purity > 95%) (Cat.SMB00072), the dye to detect intracellular ROS levels, 2′,7′-dichlorodihydrofluorescein diacetate (DCFH-DA) was purchased from Sigma-Aldrich (St. Louis, MO, USA). Dulbecco’s modified Eagle’s medium (DMEM), fetal bovine serum (FBS), and other cell culture reagents were obtained from Gibco Life Technologies (Grand Island, NY, USA). The kits for the determination of superoxide dismutase (SOD), catalase (CAT), and malondialdehyde (MDA) were provided by Nanjing Jiancheng Bioengineering Institute (Nanjing, China). Primary antibodies against NQO1, HO-1, Nrf2, β-actin and Lamin B were obtained from Abcam (Cambridge, UK). Lipofectamine^®^ RNAiMAX Transfection Reagent was obtained from Invitrogen Life Technologies (Grand Island, NY, USA). 3-(4,5-dimethylthiazol-2-yl)-2,5-diphenyltetrazolium bromide (MTT), and all other chemicals were purchased from Sigma (St. Louis, MO, USA).

### 4.2. Cell Culture

HUVECs were purchased from Shanghai Institute of Cell Biology, Chinese Academy of Sciences (Shanghai, China), and cultured in DMEM supplemented with 10% heat-inactivated FBS, 100 μg/mL penicillin and 100 U/mL streptomycin. The cells were maintained at 5 × 10^6^ cells/dish in 100 mm dishes and incubated at 37 °C in a humidified atmosphere containing 5% CO_2_.

### 4.3. MTT Assay

Cell viability was determined by MTT assay. HUVECs were seeded at a density of 5 × 10^3^ cells/well into 96-well plates. After cells were subjected to different treatments, they were incubated with MTT solution at a final concentration of 0.5 mg/mL for 4 h at 37 °C. Next, the medium was removed and DMSO (150 μL) was added to each well. Optical density was measured at an absorption wavelength of 570 nm with a microplate reader (Bio-Tek, Winooski, VT, USA). Cell survival radio was expressed as a percentage of the control.

### 4.4. ROS Measurement

For measurement of ROS, HUVECs (1 × 10^6^ cells/well in 6-well plates) were pre-treated with of without increasing concentrations of SAL (0.1, 1, 10 μM) for 24 h. Oxidative stress was induced by the addition of H_2_O_2_ (300 μM) for another 4 h. After removing the culture supernatant and washing with phosphate-buffered saline (PBS), the cells were stained with 10 μM DCFH-DA in serum free medium for 30 min in the dark. Then we washed the cells three times with PBS. Fluorescence intensity was recorded at an excitation wavelength of 488 nm and an emission wavelength of 525 nm by using a Synergy TM 4 Multi-Mode Microplate Reader (Bio-Tek).

### 4.5. SOD, CAT and MDA Measurements

HUVECs (5 × 10^3^ cells/well in 96-well plates) were pre-treated with or without increasing concentrations of SAL (0.1, 1, 10 μM) for 24 h, and incubated with 300 μM H_2_O_2_ for another 4 h. Then, the cellular SOD, CAT and MDA were measured with corresponding assay kits (Jiancheng Bioengineering Institute, Nanjing, China) according to the manufacturer’s protocol. The optical density of SOD, CAT and MDA were measured at absorption wavelengths of 450 nm, 405 nm, and 532 nm, respectively, with a microplate reader (Bio-Tek). Levels of SOD, CAT and MDA were calculated and normalized to the normal control.

### 4.6. Western Blotting

Cells were treated as described above, washed with PBS twice, lysed, harvested and pelleted by centrifugation at 12,000 rpm for 15 min at 4 °C. The supernatants were collected and stored at −80 °C until use. The protein concentration was determined using BCA protein assay kits (Thermo Scientific, Hudson, NH, USA). After the addition of sample loading buffer, an equal amount of protein from each sample was resolved 10% SDS-polyacrylamide gel electrophoresis and then electrophoretically transferred onto polyvinylidene difluoride membranes (Millipore, Bedford, MA, USA) at 500 mA for 1 h. The membranes were blocked with 5% skimmed milk for 1 h, followed by incubation with primary anti-NQO1, anti-HO-1, anti-Nrf2, anti-actin, or anti-lamin B antibodies (1:2000 dilution) at 4 °C overnight. Then, membranes were washed three times with TBST and incubated with horseradish peroxidase-conjugated goat anti-rabbit or anti-mouse antibodies (1:1000 dilution) for 1 h at 37 °C. After being washed 5 min，five times with TBST again, bolts were detected using ChemiDoc™ XRS+ System (Bio-Rad, Hercules, CA, USA) with enhanced chemiluminescence (ECL) detection regent (Millipore).

### 4.7. Real-Time Polymerase Chain Reaction (RT-PCR)

After the intervention, we washed the treated cells twice with PBS, and the total RNA of HUVECs was extracted with Trizol reagent (Invitrogen, Carlsbad, CA, USA). Then, following the manufacturer’s protocol, we converted RNA to cDNA by using a PrimeScript RT reagent kit (Takara Bio, Shiga, Japan). Real-time qPCR was performed, using a SYBR Green system (Applied Biosystems, Foster City, CA, USA) and an ABI 7500 (Applied Biosystems). All genes’ mRNA expression was normalized to the housekeeping gene β-actin. The primer sequences were as follows:

Nrf2: Forward primer, 5′-CATCCAGTCAGAAACCAGTGG-3′;
Reverse primer, 5′-GCAGTCATCAAAGTACAAAGCAT-3′;
HO-1: Forward primer, 5′-CTTCTTCACCTTCCCCAACA-3′;
Reverse primer, 5′-ATTGCCTGGATGTGCTTTTC-3′;
NQO1: Forward primer, 5′- GGGATCCACGGGGACATGAATG-3′;
Reverse primer, 5′-ATTTGAATTCGGGCGTCTGCTG-3′;
β-actin: Forward primer, 5′-GGAAATCGTGCGTGACATTA-3′;
Reverse primer, 5′-GGAGCAATGATCTTGATCTTC-3′;

The fold change between groups were calculated by using the Ct value by the method 2^−ΔΔCt^ (ΔCt = Ct [target gene] − Ct[β-actin]).

### 4.8. Nrf2-siRNA Transient Transfection

HUVECs were transiently transfected with Nrf2 siRNA by using Lipofectamine^®^ RNAiMAX Transfection Reagent according to the manufacturer’s instructions. Cells were used in experiments 48 h after transfection. The successful knockdown was confirmed by using RT-PCR.

### 4.9. Preparation of Nuclear and Cytosolic Fractions

Nuclear and Cytosolic Fractions were performed according to manufacturer’s instruction (Pierce Biotechnology, Rockford, IL, USA). After treatment, HUVECs were harvested, washed with PBS, and centrifuged at 500× *g* for 3 min. Then, we removed and discarded the supernatant, added ice-cold CER I to the cell pellet, and fully suspended the cell pellet. After incubation on ice for 10 min, cells were added with ice-cold CERII, suspended and centrifuged at 16,000× *g* for 5 min. We immediately transferred the supernatant (cytoplasmic extract) to a clean pre-chilled tube, and placed this tube on ice until storage. The insoluble fraction was suspended in ice-cold NER, vortex for 15 s every 10 min for a total 40 min, and centrifuged at 16,000× *g* for 10 min. We, again, immediately transferred the supernatant (nuclear extract) to a clean pre-chilled tube, and placed this tube on ice until storage. Finally, we stored the extracts at −80 °C until use.

### 4.10. Immunofluorescence Staining

Immunofluorescence staining was performed when the cells reached 80% confluence. After treatment, HUVECs were fixed in 4% paraformaldehyde for 20 min, rehydrated in PBS for 15 min, and immersed in 0.1% TritonX-100 for 30 min, at room temperature. After being washed twice with PBS, the fixed and permeabilized cells were blocked with 5% Goat serum blocking fluid in TBST for 1 h, and then incubated with the primary anti-Nrf2 antibody (1:200 dilution) at 4 °C overnight, followed by another incubation with the polyclonal Alexa Fluor^®^ 488 goat anti-rabbit IgG (1:200 dilution) for 1 h at room temperature. Cellular nuclei were stained with DAPI (1:1000 dilution). The images of Nrf2 with Alexa Fluor^®^ 488 staining were randomly chosen and analyzed with a confocal scanning laser microscope Zeiss LSM 710 (Carl Zeiss Microscopy GmbH, Jena, Germany).

### 4.11. Statistical Analysis

Statistical analysis was performed using GraphPad Prism software version 6.0 (GraphPad Software Inc., San Diego, CA, USA). One-way ANOVA was used to compare three or more groups. All results are expressed as mean ± SD. *p* < 0.05 was considered to be statistically significant.
